# Self-Efficacy as a Mediator Between Lifestyle and Self-Perceptions of Aging

**DOI:** 10.1093/geroni/igaa057.1367

**Published:** 2020-12-16

**Authors:** Andrew Steward, Leslie Hasche

**Affiliations:** University of Denver, Denver, Colorado, United States

## Abstract

A growing body of literature demonstrates the public health impacts of negative self-perceptions of aging (SPA). However, minimal research has explored mediating mechanisms as well as which lifestyle activities may influence SPA. Based on theory and prior research in successful aging, this study explored the impact of lifestyle activities on SPA and tested self-efficacy as a mediator between lifestyle activities and SPA. This study analyzed cross-sectional data from the psychosocial module in the 2018 wave of the Health and Retirement study (N = 2,675; mean age = 65.67/ SD = 9.95). Eight SPA items (e.g., “Things keep getting worse as I get older”) were derived from the Attitudes Toward Own Aging subscale of the Philadelphia Geriatric Center Morale Scale. 10 items measure self-efficacy on a 6-point Likert-type scale from strongly disagree to strongly agree. 21 lifestyle activities covering physical, intellectual, social, and spiritual domains were dichotomized into whether respondents participate at least monthly. Multiple regression analyses were utilized. Results indicate that self-efficacy explained 23% of the variance in SPA while holding constant age, gender, and functional ability. Sobel’s test showed that self-efficacy mediated the relationship between lifestyle activities and SPA. Exercise explained the most variance in SPA, followed by computer use and volunteering. Building upon successful aging literature, this study demonstrates the impact of self-efficacy and helps distinguish which lifestyle activities may be most effective in improving SPA. In addition to individual-level lifestyle activities, the impact of structural interventions on SPA should be tested in future research.

